# Prospective associations of meat consumption during childhood with measures of body composition during adolescence: results from the GINIplus and LISAplus birth cohorts

**DOI:** 10.1186/s12937-016-0222-5

**Published:** 2016-12-05

**Authors:** Carla Harris, Anette Buyken, Andrea von Berg, Dietrich Berdel, Irina Lehmann, Barbara Hoffmann, Sibylle Koletzko, Berthold Koletzko, Joachim Heinrich, Marie Standl

**Affiliations:** 1 Institute of Epidemiology I, Helmholtz Zentrum München – German Research Centre for Environmental Health, Ingolstädter Landstr. 1, 85764 Neuherberg, Germany; 2 DONALD Study, IEL – Nutritional Epidemiology, University of Bonn, Dortmund, Germany; 3 Research Institute, Department of Pediatrics, Marien-Hospital, Wesel, Germany; 4 Core Facility ‘Studies’, Helmholtz Zentrum für Umweltforschung UFZ, Leipzig, Germany; 5 Department of Environmental Immunology, Helmholtz Zentrum für Umweltforschung UFZ, Leipzig, Germany; 6 Institute of Occupational, Social and Environmental Medicine, Heinrich-Heine University of Düsseldorf, Düsseldorf, Germany; 7 Ludwig Maximilians-Universität München, Dr. von Hauner Children’s Hospital, Munich, Germany; 8 Institute and Outpatient Clinic for Occupational, Social and Environmental Medicine, Inner City Clinic, University Hospital of Munich (LMU), Munich, Germany

**Keywords:** Meat intake, Body composition, Adolescence, Protein, Longitudinal study, Fat mass, Fat free mass

## Abstract

**Background:**

Higher meat and protein intakes have been associated with increased body weight in adults, but studies evaluating body composition are scarce. Furthermore, our knowledge in adolescents is limited. This study aimed to investigate the prospective associations of intakes of different meat types, and their respective protein contents during childhood, with body composition during adolescence.

**Methods:**

Dietary (using food frequency questionnaires) and body composition (measured by bioelectrical impedance) data were collected from the 10- and 15-year follow-up assessments respectively, of the GINIplus and LISAplus birth cohort studies. Sex-stratified prospective associations of meat and meat protein intakes (total, processed, red meat and poultry) with fat mass index (FMI) and fat free mass index (FFMI), were assessed by linear regression models (*N* = 1610).

**Results:**

Among males, higher poultry intakes at age 10 years were associated with a higher FMI at age 15 years [β = 0.278 (SE = 0.139), *p* = 0.046]; while higher intakes of total and red meat were prospectively associated with higher FFMI [0.386 (0.143), *p* = 0.007, and 0.333 (0.145), *p* = 0.022, respectively]. Additionally in males, protein was associated with FFMI for total and red meat [0.285 (0.145) and 0.356 (0.144), respectively].

**Conclusions:**

Prospective associations of meat consumption with subsequent body composition in adolescents may differ by sex and meat source.

**Electronic supplementary material:**

The online version of this article (doi:10.1186/s12937-016-0222-5) contains supplementary material, which is available to authorized users.

## Background

Concerns regarding excessive meat intake include increased risks of all-cause mortality [1], cancer [2], CVD [3] and diabetes mellitus [4]. Observational studies have also associated high meat intakes with increased risk of weight gain and obesity [5]. Red and processed meats in particular, have been associated with increased weight gain. However, meat types are very diverse, and differ substantially from each other in terms of macronutrient and energy composition as well as processing. A number of observational studies have reported animal protein, the main macronutrient component of meat, to be directly associated with weight gain [6]. On the other hand, animal protein is known to increase satiety and thermogenesis [7], and intervention studies have reported beneficial effects of high protein diets on fat loss and weight maintenance [8]. Amino acids obtained from meat protein have been proposed to exert an anabolic effect on muscle mass, and may be important in the development and maintenance of lean tissue [9]. It is hence possible that the associations reported between meat intake and weight gain in observational studies could be due to gains in lean mass rather than fat mass. Indeed, positive prospective associations between animal protein intake and lean body mass from puberty to young adulthood have been reported in females [10].

A better understanding of the role of different meat types and their respective protein contents is needed in order to shed light on the underlying factors driving associations between meat intake and weight gain. Furthermore, the evaluation of body composition can determine whether weight gains associated with meat intake are a result of accumulating fat mass, fat-free mass, or both. Hence, in order to appreciate the true role of meat intake in adiposity, accurate body composition data are necessary.

In a large proportion of German adolescents, meat intakes exceed recommended amounts [11], and the prevalence of overweight and obesity is high and rising further [12]. Considering that overweight in adolescence is known to track into adulthood [13], the identification of meat as a contributor towards increased fat mass in adolescence could have important implications for the early prevention of overweight and associated comorbidities. There is a need for longitudinal studies on the association between meat intake and body composition during adolescence, a critical life stage during which fast weight-gain occurs [14]. The aims of the present study were thus to investigate prospective associations of the consumption of different sources of meat and meat-protein during childhood, with fat mass and fat-free mass during adolescence.

## Methods

### Subjects

The present study used data from the 10- and 15-year follow-up assessments of the ongoing GINIplus (*G*erman *I*nfant *N*utritional *I*ntervention *plus* environmental and genetic influences on allergy development) and LISAplus (Influence of *L*ifestyle-Related Factors on the *I*mmune *S*ystem and the Development of *A*llergies in Childhood *plus* the Influence of Traffic Emissions and Genetics) birth cohort studies. Healthy full-term new-borns were recruited from obstetric clinics within four German cities between 1995 and 1999. Information was collected using identical questionnaires and at physical examinations. The study designs, recruitment and exclusion criteria have been described previously [15, 16]. For both studies, approval by the local ethics committees (Bavarian Board of Physicians, University of Leipzig, Board of Physicians of North-Rhine-Westphalia) and written consent from participant’s families were obtained.

### Exposure variables

Dietary intake data was obtained from the 10-year follow-up assessment, using a self-administered food frequency questionnaire (FFQ), designed and validated to assess food and nutrient intake over the past year in school-aged children [17]. In brief, subjects were asked to report estimated frequency and portion size of intakes of 80 food items. A quality control procedure was applied based on recommendations by Willett et al. for data cleaning in nutritional epidemiology [18].

Four meat types were defined: *processed meat* (salami, liver sausage, cold meat, bratwurst and wiener- or pork-sausage), *red meat* (pork, beef, veal), *poultry* (any poultry meat) and *other meats* (offal and ready meals with meat). The protein content (g/day) of each of the different meat types was calculated based on the German Food Code and Nutrient Database (BLS) version II.3.1 [19], and converted to kcal/d (g/d multiplied by 4). The daily intakes (mg/d) of essential amino acids (EAA), saturated fatty acids (SFA), monounsaturated fatty acids (MUFA) and polyunsaturated fatty acids (PUFA) were also obtained from the FFQ by use of the same database. Total meat intake (the sum of all meat types) and each individual meat type, as well as their respective protein contents, were included as exposures in the statistical analyses. The food-group “other meats” was rarely consumed and was not individually analysed.

### Outcome variables

Measures of fat mass and fat free mass were obtained during the 15-year physical examination by means of phase sensitive bioelectrical impedance (BIA). Fat mass index (FMI) and fat-free mass index (FFMI) were calculated by dividing fat mass and fat-free mass (kg), respectively, by height squared (kg/m^2^) measured without shoes at the same examination. Blood samples were also obtained from willing participants during the 10- and 15-year follow-up physical examinations. The concentrations (mmol/L) of total cholesterol, LDL, HDL, and triglycerides (TAG) were measured in serum using homogenous enzymatic colorimetric methods on a Modular Analytics System from Roche Diagnostics GmbH Mannheim according to the manufactures instructions. External controls were used in accordance with the guidelines of the German Society of Clinical Chemistry and Laboratory Medicine. The ratio of total to HDL cholesterol (TOTAL:HDL) was calculated by dividing total cholesterol by HDL.

### Adjustment variables

Statistical models were adjusted for study (GINI observation arm; GINI intervention arm; LISA), recruitment region (Munich; Wesel; Bad Honnef; Leipzig), parental education level (highest level achieved by mother or father: ≤10^th^grade = low/medium; >10^th^grade = high), exact age at BIA measurement (years), sedentary behaviour at age 15 years (≤2 h screen time/day = low; > 2 h screen time/day = high), pubertal onset (any presence of acne or spots, pubic or axillary hair, breast development, menstruation, penis or testicle enlargement at age 10 years: yes; no), and weight category at age 10 years (BMI z-score ≤ 1 = normal weight; BMI z-score > 1 = overweight). BMI z-scores used to categorize body weight were calculated according to the 2007 BMI-for-age WHO growth reference for school-aged children and adolescents [20]. Due to non-random loss-to follow-up, children with low parental education were underrepresented in our study population (Additional file 1: Table S1), therefore low (<10^th^grade) and medium (10^th^grade) parental education were combined into low/medium.

### Statistical analysis

Subjects providing complete data for outcome, exposure and adjustment variables, were included (*N* = 1736). Participants were excluded if they reported an illness affecting diet at 10 or 15 years (e.g. diabetes, anorexia, coeliac disease, cancer) or medical dietary indications, such as gluten-free or lactose-free diets, at age 15 years (*n* = 82). Clear outliers in outcomes (*n* = 2) and exposures (*n* = 42) were visually identified using descriptive plots and excluded from the analyses (Additional file 2: Figure S1). Meat and meat protein intake variables were adjusted for daily caloric intake using the nutrient residual model. For this we computed sex-specific residuals from a regression model where meat and protein variables (kcal/day) were regressed on energy intake (kcal/day) at age 10 years. As these residuals are uncorrelated with total energy intake the variation due to the nutrient composition of the diet, rather than the combination with total amount of food, can be evaluated. Due to non-linearity, residuals were categorized into sex-specific tertiles (T1 = low, T2 = medium and T3 = high intake).

Main subject characteristics for the total study population, and stratified by energy-adjusted meat intake tertiles, were described by medians (25^th^ percentile; 75th percentile) or counts (%). Differences between meat intake tertiles were tested using Kruskal-Wallis test for continuous variables and χ^2^-test for categorical variables. All statistical analyses were performed and presented stratified by sex. Prospective associations of consumption of meat (total meat, processed, red meat, poultry) and meat protein (total meat protein, processed meat protein, red meat protein, poultry protein) at age 10 years with FMI and FFMI at age 15 years, were assessed by linear regression models. First, minimally adjusted models (MIN) were fit, adjusting for study, recruitment region, parental education level, pubertal onset, age at BIA measurement and sedentary behaviour. As significant associations between meat intake and BMI at age 10 years have been previously reported [21], main models (MAIN) were fit separately, further adjusting for weight category at age 10 years. We performed additional analyses where we further adjusted the main model for EAA, SFA, MUFA or PUFA, respectively. These variables were included in the model as energy-adjusted residuals (computed as described above for meat and protein residuals). We also tested for possible interactions by including an interaction term between the meat or protein exposures and weight category, following which stratified analyses (normal weight; overweight) were performed. Finally, we repeated our main analyses using blood lipid parameters as secondary outcomes in a subgroup of the study population who provided measurements at ages 10 and 15 years (*n* = 1309). Linear regression models were used to assess the prospective associations of consumption of meat and meat protein at age 10 years with changes in blood lipids (∆LDL, ∆HDL, ∆TAG and ∆TOTAL:HDL) from age 10 to 15 years. Models were adjusted as in the previously described main model, with further adjustment for the respective blood lipid measurement at age 10 years.

Results are presented as β-coefficients (β), along with their standard errors (SE) with reference to the lowest intake tertile (T1). Meat intake residual coefficients have an isocaloric substitution interpretation. A two-sided α-level of 5% was considered significant. For the stratified analyses, we corrected for multiple testing using Bonferroni correction, yielding a corrected two-sided alpha level of 0.025 (0.05/2 = 0.025). Since the meat group “poultry” was composed by only one food item (poultry meat), each poultry tertile includes the same subjects as its respective poultry protein tertile; therefore, the calculated regression coefficients for poultry are identical for both meat and meat protein intakes and are hence only reported when referring to meat intakes. All analyses were conducted using R (www.r-project.org), version 3.2.2 [22].

## Results

### Study population

Data from 1610 participants (797 females and 813 males) were included in the analyses (Figure S1). Descriptive characteristics are displayed in Table 1. At age 10 years, 16.7% females and 22.5% males were overweight according to WHO cut-off criteria (10.3 and 10.8%, respectively, according to IOTF cut-offs [23]). Children in the highest meat intake tertile were significantly more likely to be overweight at age 10 years. Most children in the study population were from Munich and from families with high parental education.

**Table 1:** Basic characteristics of study population by tertiles of total meat intake at age 10 years

	Females					Males				
	Total meat (*n* = 797)	Total meat tertiles				Total meat (*n* = 813)	Total meat tertiles			
		T1 (*n* = 266)	T2 (*n* = 266)	T3 (*n* = 265)	*p*-val^a^		T1 (*n* = 271)	T2 (*n* = 271)	T3 (*n* = 271)	*p*-val^a^
10 years										
BMI (kg/m^2^)	16.7 (15.5; 18.3)	16.4 (15.5; 17.9)	16.9 (15.5; 18.3)	16.9 (15.5; 18.6)	**0.037**	16.7 (15.6; 18.4)	16.5 (15.4; 18.3)	16.7 (15.5; 18.1)	16.9 (15.9; 18.9)	**0.030**
Overweight, *n* (%)^b^	133 (16.7)	32 (12)	44 (16.5)	57 (21.5)	**0.014**	183 (22.5)	54 (19.9)	54 (19.9)	75 (27.7)	**0.045**
Age (years)	10.7 (10.5; 11.2)	10.7 (10.5; 11.2)	10.8 (10.5; 11.2)	10.7 (10.4; 11.1)	0.162	10.7 (10.4; 11.1)	10.7 (10.4; 11.1)	10.7 (10.4; 11)	10.7 (10.4; 11.1)	0.923
Sedentary behaviour [high]^c^, *n* (%)	65 (8.2)	15 (5.7)	23 (8.7)	27 (10.3)	0.149	103 (12.8)	37 (13.7)	31 (11.6)	35 (13.1)	0.759
Pubertal onset [Yes]^d^, *n* (%)	366 (45.9)	117 (44)	120 (45.1)	129 (48.7)	0.526	81 (10)	30 (11.1)	25 (9.2)	26 (9.6)	0.750
15 years										
BMI (kg/m^2^)	20.3 (18.8; 22.1)	20.1 (18.6; 21.6)	20.4 (19.1; 22.3)	20.4 (18.8; 22.5)	0.066	19.9 (18.5; 21.9)	19.6 (18.2; 21.5)	19.8 (18.3; 21.6)	20.4 (18.9; 22.6)	0.001
Overweight, *n* (%)^b^	105 (13.2)	22 (8.3)	38 (14.3)	45 (17)	**0.010**	151 (18.6)	46 (17)	43 (15.9)	62 (22.9)	0.078
Fat mass index (kg/m^2^)	5.5 (4.6; 6.6)	5.2 (4.5; 6.2)	5.6 (4.6; 6.7)	5.8 (4.7; 6.9)	0.008	3.6 (2.8; 4.7)	3.5 (2.8; 4.5)	3.5 (2.7; 4.5)	3.8 (2.8; 5.1)	0.028
Fat free mass index (kg/m^2^)	14.9 (13.8; 15.8)	14.8 (13.8; 15.5)	14.9 (13.8; 16)	14.9 (13.8; 15.9)	0.411	16.3 (15.3; 17.6)	16 (15.2; 17.3)	16.3 (15.2; 17.4)	16.6 (15.6; 18)	0.002
Age (years)	15.2 (15; 15.3)	15.2 (15; 15.3)	15.2 (15.1; 15.3)	15.1 (15; 15.3)	0.336	15.1 (15; 15.3)	15.2 (15; 15.3)	15.1 (15; 15.3)	15.1 (15; 15.3)	0.704
Sedentary behaviour [high]^c^, *n* (%)	386 (48.4)	116 (43.6)	134 (50.4)	136 (51.3)	0.152	522 (64.2)	172 (63.5)	166 (61.3)	184 (67.9)	0.260
Basis characteristics										
Study										
GINI control, *n* (%)	282 (35.4)	92 (34.6)	103 (38.7)	87 (32.8)	0.226	258 (31.7)	82 (30.3)	91 (33.6)	85 (31.4)	0.862
GINI intervention, n (%)	254 (31.9)	86 (32.3)	89 (33.5)	79 (29.8)		238 (29.3)	85 (31.4)	74 (27.3)	79 (29.2)	
LISA, *n* (%)	261 (32.7)	88 (33.1)	74 (27.8)	99 (37.4)		317 (39)	104 (38.4)	106 (39.1)	107 (39.5)	
Region										
Munich, *n* (%)	417 (52.3)	154 (57.9)	137 (51.5)	126 (47.5)	0.094	416 (51.2)	138 (50.9)	142 (52.4)	136 (50.2)	0.960
Leipzig, *n* (%)	69 (8.7)	22 (8.3)	18 (6.8)	29 (10.9)		79 (9.7)	24 (8.9)	29 (10.7)	26 (9.6)	
Bad Honef, *n* (%)	34 (4.3)	14 (5.3)	10 (3.8)	10 (3.8)		40 (4.9)	15 (5.5)	11 (4.1)	14 (5.2)	
Wesel, *n* (%)	277 (34.8)	76 (28.6)	101 (38)	100 (37.7)		278 (34.2)	94 (34.7)	89 (32.8)	95 (35.1)	
Parental educ. [High], *n* (%)^e^	578 (72.5)	205 (77.1)	193 (72.6)	180 (67.9)	0.062	552 (67.9)	192 (70.8)	182 (67.2)	178 (65.7)	0.415

Values are medians for continuous variables (25th percentile; 75th percentile) and *n* (%) for categorical variables. ^a^Differences between tertiles were tested by Kruskal-Walis test for continuous variables and X^2^-test for categorical variables; ^b^BMI z-score > 1; ^c^Hours spent on screen activities > 2; ^d^Presence of any sign of pubertal onset; ^e^Highest level achieved by mother or father > 10y. Significant *p*-values marked in bold

### Regression analyses

#### Primary outcomes (FMI and FFMI)

Results of the minimally adjusted (MIN) and main (MAIN) linear regression models are presented in Table 2. In females, the MIN models showed that high (T3) total meat and poultry intakes at age 10 years were related to higher FMI at 15 years (*p*-value for linear trend = 0.006 and 0.019, respectively). These associations were no longer significant in the MAIN models. Similar results were observed for protein intakes in females. In males, the MIN models indicated that high (T3) poultry intakes at age 10 years were associated with higher FMI at age 15 years, and high (T3) red and processed meat intakes were related to higher FFMI; while high (T3) total meat intakes were related to both higher FMI and higher FFMI. Following further adjustment for BMI category in the MAIN model, high (T3) poultry intake at age 10 years remained significantly associated with higher FMI at age 15 years [0.278 (0.139)] (*p*-value for linear trend = 0.047), while high (T3) total and red meat intakes at age 10 years were significantly associated with higher FFMI at age 15 years [0.386 (0.143) and 0.333 (0.145), respectively] (*p*-value for linear trend = 0.007 and 0.022, respectively). Similar associations were observed with the respective protein intakes of all meat types [0.285 (0.145) for high total meat protein with higher FFMI, and 0.356 (0.144) for high red meat protein with higher FFMI].

**Table 2:** Prospective association of tertiles of meat and meat protein intakes with FMI and FFMI

	FMI							FFMI						
	T2 vs T1			T3 vs T1				T2 vs T1			T3 vs T1			
	β	SE	*p*-val	β	SE	*p*-val	p-trend	β	SE	*p*-val	β	SE	*p*-val	p-trend
Females														
Total meat														
MIN	0.255	0.149	0.088	0.411	0.150	**0.006**	**0.006**	0.108	0.130	0.405	0.106	0.130	0.418	0.418
MAIN	0.177	0.130	0.174	0.224	0.131	0.088	0.088	0.053	0.119	0.658	−0.028	0.120	0.818	0.818
Processed														
MIN	0.077	0.149	0.604	0.265	0.150	0.077	0.077	0.209	0.129	0.105	0.164	0.130	0.208	0.206
MAIN	0.005	0.130	0.967	0.194	0.130	0.137	0.138	0.159	0.118	0.180	0.113	0.119	0.340	0.338
Red meat														
MIN	−0.178	0.150	0.236	0.095	0.152	0.531	0.544	0.080	0.130	0.539	0.038	0.132	0.776	0.771
MAIN	−0.116	0.131	0.374	−0.063	0.133	0.636	0.627	0.124	0.119	0.297	−0.076	0.121	0.533	0.550
Poultry														
MIN	−0.104	0.149	0.487	0.355	0.150	**0.018**	**0.019**	−0.122	0.130	0.349	0.115	0.131	0.378	0.380
MAIN	−0.146	0.130	0.260	0.254	0.131	0.052	0.053	−0.152	0.119	0.202	0.044	0.120	0.714	0.716
Total meat protein														
MIN	0.067	0.150	0.653	0.419	0.152	**0.006**	**0.006**	0.094	0.130	0.471	0.088	0.132	0.503	0.502
MAIN	−0.013	0.131	0.922	0.246	0.133	0.064	0.065	0.037	0.119	0.758	−0.035	0.121	0.772	0.773
Processed (Protein)														
MIN	0.105	0.149	0.482	0.226	0.150	0.131	0.131	0.011	0.129	0.933	0.233	0.130	0.073	0.074
MAIN	0.077	0.130	0.552	0.077	0.131	0.558	0.555	−0.009	0.118	0.943	0.128	0.119	0.283	0.287
Red meat (Protein)														
MIN	−0.113	0.150	0.453	0.100	0.153	0.512	0.522	0.079	0.130	0.543	0.014	0.132	0.915	0.909
MAIN	−0.088	0.131	0.503	−0.052	0.133	0.696	0.690	0.097	0.119	0.415	−0.094	0.121	0.438	0.450
Poultry (protein)^a^														
MIN	–	–	–	–	–	–	–	–	–	–	–	–	–	–
MAIN	–	–	–	–	–	–	–	–	–	–	–	–	–	–
Males														
Total meat														
MIN	−0.084	0.162	0.604	0.395	0.162	**0.015**	**0.015**	0.032	0.162	0.846	0.552	0.162	**0.001**	**0.001**
MAIN	−0.096	0.138	0.489	0.213	0.139	0.124	0.125	0.021	0.143	0.884	0.386	0.143	**0.007**	**0.007**
Processed														
MIN	0.076	0.164	0.641	0.232	0.163	0.155	0.155	0.049	0.164	0.763	0.398	0.163	**0.015**	**0.015**
MAIN	0.096	0.139	0.493	0.095	0.139	0.495	0.495	0.067	0.144	0.641	0.273	0.144	0.057	0.057
Red meat														
MIN	−0.029	0.163	0.858	0.166	0.164	0.312	0.316	0.182	0.163	0.264	0.433	0.165	**0.009**	**0.009**
MAIN	−0.031	0.139	0.826	0.057	0.140	0.686	0.690	0.181	0.143	0.207	0.333	0.145	**0.022**	**0.022**
Poultry														
MIN	−0.018	0.162	0.914	0.418	0.163	**0.010**	**0.011**	−0.082	0.164	0.617	0.159	0.164	0.333	0.336
MAIN	−0.041	0.139	0.766	0.278	0.139	**0.046**	**0.047**	−0.104	0.144	0.471	0.028	0.144	0.844	0.849
Total meat protein														
MIN	−0.045	0.163	0.783	0.412	0.164	**0.012**	**0.012**	0.080	0.164	0.626	0.479	0.164	**0.004**	**0.004**
MAIN	−0.007	0.139	0.960	0.202	0.140	0.151	0.153	0.115	0.144	0.426	0.285	0.145	**0.050**	**0.050**
Processed (Protein)														
MIN	−0.015	0.163	0.929	0.312	0.163	0.055	0.055	−0.092	0.163	0.575	0.376	0.163	**0.021**	**0.021**
MAIN	0.043	0.139	0.758	0.131	0.139	0.346	0.346	−0.039	0.144	0.786	0.211	0.144	0.143	0.144
Red meat (Protein)														
MIN	−0.107	0.163	0.509	0.162	0.164	0.323	0.328	0.072	0.163	0.660	0.442	0.164	**0.007**	**0.007**
MAIN	−0.045	0.139	0.747	0.069	0.140	0.623	0.627	0.129	0.143	0.367	0.356	0.144	**0.014**	**0.014**
Poultry (protein)^a^														
MIN	–	–	–	–	–	–	–	–	–	–	–	–	–	–
MAIN	–	–	–	–	–	–	–	–	–	–	–	–	–	–

MIN: adjusted for study, region, age at BIA measurement, parental education, pubertal onset10; MAIN: MIN model further adjusted for overweight at 10y; *p*-val: *p*-value from linear regression; p-trend: *p*-value indicating linear trend. Significant *p*-values marked in bold

^a^Estimates for poultry protein not presented, as categories for protein were identical to those for poultry meat, and hence estimates are also identical

Results from the further adjusted models (adjusted for EAA, SFA, MUFA or PUFA) are presented in Additional file 3: Tables S3a for females and S3b for males. In females, additional adjustment for MUFA or PUFA resulted in significant positive associations between high (T3) total meat and meat protein intakes with FMI. When adjusting for PUFA, high (T3) poultry intakes were also significantly associated with FMI. In males, when adjusting for EAA, SFA, MUFA or PUFA, the association between high poultry intake and FMI no longer reached statistical significance (except with adjustment for MUFA, where it was weakened but remained borderline significant). The associations between red meat, total meat protein and red meat protein with FFMI in males were no longer significant following adjustment for EAA, while the association of total meat with FFMI was weakened. On the other hand, when adjusting for SFA, an additional positive association was observed between high (T3) processed meat and FFMI. When adjusting for MUFA or PUFA, the association between total meat protein intakes with FFMI was no longer significant.

#### Stratified analyses (normal weight/ overweight)

**Fig. 1:**
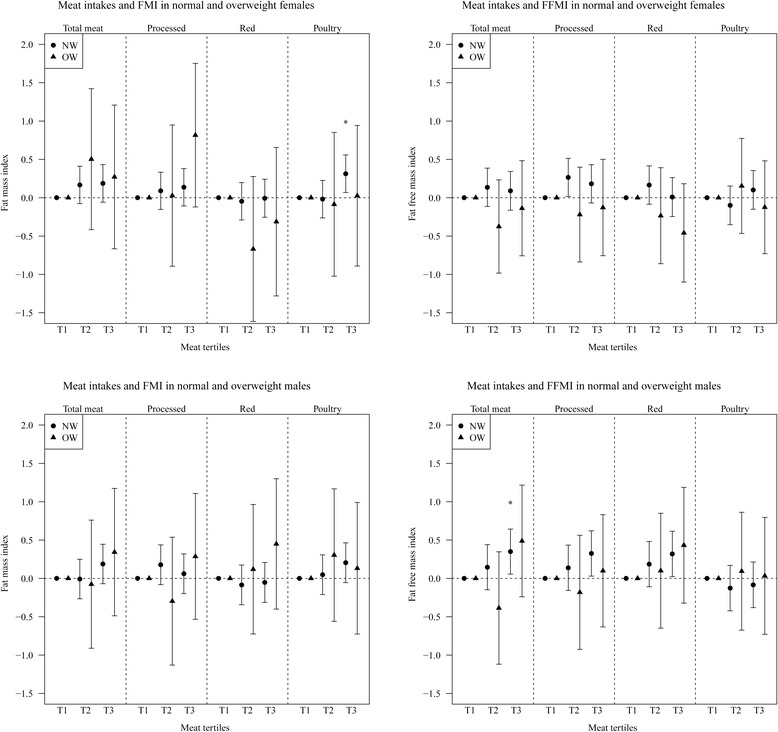
Prospective association of tertiles of meat intakes (T2 and T3 vs T1) with FMI (left) and FFMI (right), stratified by normal weight (NW) and overweight (OW) subjects in females (top) and males (bottom). *P value < 0.025 (significant after correction for multiple testing)

Stratified analyses results are presented in Fig. 1 (exact values in Additional file 4: Tables S2a for females and S2b for males). In females, high (T3) intakes of poultry in children with normal weight at age 10 years were related to higher FMI at age 15 years [0.314 (0.125)]. In males high (T3) total meat intakes in normal weight children at age 10 years was related to higher FFMI at age 15 years [0.350 (0.150)].

#### Secondary outcomes (∆LDL, ∆HDL, ∆TAG and ∆TOTAL:HDL)

Blood samples at both age 10 and 15 years were available in a subsample of 1309 participants (636 females and 673 males). In males, high (T3) red meat and red meat protein intakes were associated with increasing TAG concentrations [0.131 (0.060), *p*-value = 0.030; and 0.130 (0.060), *p*-value = 0.031, respectively]. No significant associations were observed for any of the meat or meat protein types with the other blood lipid parameters (data not shown).

## Discussion

The present study aimed at assessing the associations of meat intake at the age of 10 years with later body composition during adolescence, and to determine the role of protein in such associations. Our findings suggest that a higher poultry intake during childhood in males may lead to an accumulation of body fat during adolescence. This finding is in line with the notion that higher meat intakes promote increased weight gain, proposed in a number of observational studies [5]. Amongst these, Vergnaud et al. [24] have highlighted poultry as a possible determinant of gains in weight and waist circumference in adults. Contrary to other observational studies [5], our results suggest a beneficial association between the consumption of red meats and later lean body mass in adolescent males.

Two major differences between our and many other existing observational studies should be noted. Firstly, studies on the association of meat intake with overweight typically describe changes in body weight or BMI. These measures cannot indicate possible variation in body composition. Hence, gains in BMI or body weight are not analogous to gains in body fat, and without supporting information cannot be interpreted as such. Secondly, most studies reporting associations of different meat types with overweight have been carried out in adults. Our study population consisted of children assessed over a five-year follow-up period during adolescence, from ages 10 to 15 years. We have previously reported cross-sectional associations between higher total meat intakes and increased BMI in 10-year old children from the GINIplus and LISAplus birth cohort studies [21]. Additionally, Bradlee et al. [25] reported that adolescent boys (aged 12–16) with smaller waist circumferences tended to eat less meat. Nevertheless, in view of the present study findings, it could be suggested that associations between red meat and weight gain in adolescents may reflect increased lean mass rather than fat mass in males. Additional analyses indicated that associations between total meat and FFMI were stronger in lean than in overweight subjects. This suggests that the development of lean tissue triggered by higher meat intakes occurs more readily in leaner males, although it is possible that due to the smaller number of overweight individuals, there was not sufficient power for associations in this group to reach statistical significance.

Our data indicated that the subsequent increase in lean mass observed following higher red meat intakes could be attributed to the high protein content of this meat type. These findings are not unexpected, considering that red meat protein is a rich source of essential amino acids, known to be important for the development and preservation of lean tissue [9]. Indeed, when further adjusting our analyses for dietary EAA, the associations between red meat and red meat protein with FFMI in males were no longer significant. This was not the case when adjusting for other nutrients, suggesting that EAA might be a potentially responsible component in the association of red meat with lean body mass. Our findings are consistent with intervention studies which propose that higher protein intakes contribute towards increasing lean tissue, and can be beneficial for weight loss and maintenance [26]. Most observational studies however, fail to reproduce these findings longitudinally. Some studies even propose a detrimental effect of protein, in particular animal protein, on weight gain [27]. However, measurements of body composition are also scarce in these studies. A Danish study did report that the energy intake from protein was positively related to total fat mass in 36-year-old men and women [28]. On the other hand, a prospective relation between animal protein intake during puberty and FFMI in young adulthood was reported among females (and also in males when adjusting for FMI) [10]. Furthermore, in another study, higher protein was prospectively associated with higher FFMI in overweight and lower FMI in lean girls aged 8–10 years [29]. It is however of note that despite the greater lean mass observed in males in the present study, our secondary analyses also revealed an association of red meat and red meat protein with increasing TAG levels in males. This finding supports prospective studies which have reported a link between red meat and CVD [3, 30]. Attempts to explain positive associations between meat intake and blood lipids often refer to the high SFA content of meat as the responsible component [31]. Studies have also indicated that the consumption of lean meat (low in SFA) could have beneficial effects on cardio-metabolic risk markers [32]. Nevertheless, in our analyses, adjustment for SFA did not alter the observed association between red meat and TAG (data not shown). Further research is warranted in order to evaluate the specific role of lean meat on blood lipids in adolescence. Until this area is better understood, it is unclear whether all red meats represent a healthy dietary protein source for adolescents attempting to promote lean body mass development. Furthermore, we cannot exclude that other dietary components consumed in the dietary pattern along with red meat, could have contributed to its observed association with lean body mass. Unfortunately, we were not able to look at the separate role of protein intake in the association of poultry with FMI, which would have been interesting considering it promotes changes in body composition which oppose those of red meats. Adjusting for EAA, SFA or PUFA weakened the association of poultry with FMI in males, whilst a positive association was observed in females with adjustment for PUFA. These results reflect a complex, sex-specific role of this meat subtype in fat mass accumulation, which, from the present analyses cannot be attributed to any specific nutrient.

We highlight that the present study was carried out during adolescence, a period where growth occurs at its most rapid rate since infancy, and where significant weight gain and important changes in body composition take place [14]. Furthermore our findings were limited to males, who, under the influence of testosterone, at this stage undergo a significant increase in lean body tissue [14]. This process could be enhanced by higher protein intakes; however it has been suggested that increasing protein consumption is not entirely necessary to maintain nitrogen retention, due to an increased efficiency of protein utilization at this life-stage [33]. It is hence plausible that the metabolic response to meat intake in adolescents is different to that occurring in adults [14]. Considering the evidence for increased risk of disease associated with red and processed meat in particular [2], these findings should be interpreted with caution, keeping in mind that similar findings are not necessarily expected to be observed in adults.

A major strength of the present study is that it is based on data from two large population-based birth cohorts. The large sample size allows for robust prospective analyses, lacking thus far in observational studies concerning meat consumption and body composition. Our data allows us to evaluate specific associations of meat consumption with fat and lean body mass. Although we additionally assessed associations with changes in blood lipids, we were unfortunately not able to assess blood biomarkers of obesity such as adipokines, which would have provided further insight into the biological effects of meat intake in parallel to those reflected by our anthropometric measurements. Additionally, some other limitations were also encountered. Although study sampling was primarily population-based, non-random loss-to-follow-up, often occurring in cohort studies, meant children of lower social classes were underrepresented in the present analyses, and hence findings cannot be considered representative of the study area. Furthermore, the 10-year FFQ was completed by parents alongside their children, as young children might have difficulties recalling intakes or understanding portion sizes. Nevertheless, the questionnaire produced plausible values in terms of energy intake and any misreporting was most likely detected through extensive quality control. The improved quality of the data was obtained at the expense of reducing the sample size, although the study sample remained large with no substantial loss of power. The FFQ lacks questions regarding typically vegetarian protein sources such as tofu or pulses. Therefore, the relative caloric contribution of meat intake – as used in this study – could be overestimated among children whose diets are high in vegetable protein. When excluding children following a meat-free diet at age 10 years, our results remained consistent (data not shown).

## Conclusions

In conclusion, prospective associations of meat consumption with subsequent body composition in adolescents may differ by sex and meat source. We found that in males high poultry intake is prospectively associated with increased fat mass, while red meat in males is related to higher fat free mass. Protein from red meat likely plays a major role in its association with lean mass. These findings provide important insight into the underlying changes in body composition occurring with meat and meat protein intakes during the period of pubertal development.

## Additional files

Additional file 1: Table S1. Comparison of lost-to-follow-up and not-lost-to-follow-up study participants. (PDF 173 kb)
Additional file 2: Figure S1. Study participants. (PDF 84 kb)
Additional file 3: Table S3a. Prospective association of tertiles of meat and meat protein intakes with FMI and FFMI in females *(n* = 636) adjusted for EAA, SFA, MUFA or PUFA. **Table S3b.** Prospective association of tertiles of meat and meat protein intakes with FMI and FFMI in males (*n* = 673) adjusted for EAA, SFA, MUFA or PUFA. (PDF 272 kb)
Additional file 4: Table S2a. Prospective association of tertiles of meat and meat protein intakes (T2 and T3 vs T1) with FMI and FFMI, stratified by BMI at 10y (normal weight/overweight), in females. **Table S2b.** Prospective association of tertiles of meat and meat protein intakes (T2 and T3 vs T1) with FMI and FFMI, stratified by BMI at 10y (normal weight/overweight), in males. (PDF 248 kb)

## Abbreviations

FFMI: Fat free mass index
FFQ: Food frequency questionnaire
FMI: Fat mass index
