# Supplementary material for: Prospective associations of meat consumption during childhood with measures of body composition during adolescence: results from the GINIplus and LISAplus birth cohorts
Source: Nutr J. 2016 Dec 5;15:101. doi: 10.1186/s12937-016-0222-5 (PMC5139017; doi:10.1186/s12937-016-0222-5)
Supplement: Additional file 1: Table S1. — Comparison of lost-to-follow-up and not-lost-to-follow-up study participants. (PDF 173 kb) [file 12937_2016_222_MOESM1_ESM.pdf]

**Table S1** Comparison of lost-to-follow-up and not-lost-to-follow-up study participants

| | Females | | | Males | | |
|---|---|---|---|---|---|---|
| | Lost to Follow-up | Not lost to Follow-up | p-value | Lost to Follow-up | Not lost to Follow-up | p-value |
| N | 729 | 797 | | 755 | 813 | |
| **10 years** | | | | | | |
| Age (years) | 10.8 (0.5) | 10.8 (0.5) | 0.426 | 10.9 (0.5) | 10.8 (0.5) | **0.000** |
| BMI (kg/m2) | 17.2 (2.5) | 17.1 (2.3) | 0.627 | 17.2 (2.6) | 17.2 (2.3) | 0.935 |
| Total caloric intake (kcal/d) | 18.1 (4.9) | 18.1 (4.9) | 0.836 | 21.1 (6) | 20.9 (5.7) | 0.464 |
| Overweight (%) | 19.4 | 16.7 | 0.196 | 22.8 | 22.5 | 0.902 |
| Sedentary behariour[a] [high] (%) | 9.4 | 8.2 | 0.466 | 13.8 | 12.8 | 0.600 |
| Pubertal onset [yes](%) | 47.5 | 45.9 | 0.535 | 12.6 | 10 | 0.107 |
| **15 years** | | | | | | |
| Age (years) | 15.5 (0.3) | 15.4 (0.3) | 0.156 | 15.5 (0.3) | 15.4 (0.3) | 0.050 |
| BMI (kg/m2) | 20.5 (2.9) | 20.7 (2.9) | 0.195 | 20.7 (3.3) | 20.6 (3.2) | 0.382 |
| Total caloric intake (kcal/d) | 17.5 (5.7) | 18 (5.6) | 0.142 | 23.6 (6.9) | 23.7 (6.6) | 0.803 |
| Overweight (%) | 12 | 13.2 | 0.564 | 19.5 | 18.6 | 0.681 |
| Sedentary behariour[a] [high] (%) | 46.8 | 48.4 | 0.583 | 59.5 | 64.2 | 0.069 |
| **Basic characteristics** | | | | | | |
| Study | | | | | | |
| *GINI observation* | 38.7 | 35.4 | 0.087 | 36.8 | 31.7 | 0.070 |
| *GINI intervention* | 26.7 | 31.9 | | 28.9 | 29.3 | |
| *LISA* | 34.6 | 32.7 | | 34.3 | 39 | |
| Region | | | | | | |
| *Munich* | 49.1 | 52.3 | **0.046** | 51.3 | 51.2 | **0.019** |
| *Leipzig* | 5.9 | 8.7 | | 6.2 | 9.7 | |
| *Bad Honef* | 5.1 | 4.3 | | 3.6 | 4.9 | |
| *Wesel* | 39.9 | 34.8 | | 38.9 | 34.2 | |
| Parental education[b] (%) | | | | | | |
| *Low* | 6.3 | 3.9 | **0.029** | 8.4 | 4.3 | **0.005** |
| *Medium* | 26.4 | 23.6 | | 26.6 | 27.8 | |
| *High* | 67.2 | 72.5 | | 65.1 | 67.9 | |

Lost-to-follow-up: Dietary data at age 10 years but no available body composition data at age 15 years;
Not-lost-to-follow-up: Dietary data at age 10 years and available body composition data at age 15 years (current study sample);
Categorical variables presented as percentages, tested by Fisher's exact test (variables with 2 levels), or by Pearson's Chi2 test (variables with > 2 levels);
Continuous variables presented as mean (standard deviation), tested by t-test; [a]Hours spent on screen behaviours (≤ 2 hours = low; >2 hours = high); [b]Highest level achieved by mother or father (<10 years = low; 10 years = medium; >10 years = high); Significant p-values marked in bold.
