# Supplementary figures and images for: Prospective associations of meat consumption during childhood with measures of body composition during adolescence: results from the GINIplus and LISAplus birth cohorts
Source: Nutr J. 2016 Dec 5;15:101. doi: 10.1186/s12937-016-0222-5 (PMC5139017; doi:10.1186/s12937-016-0222-5)

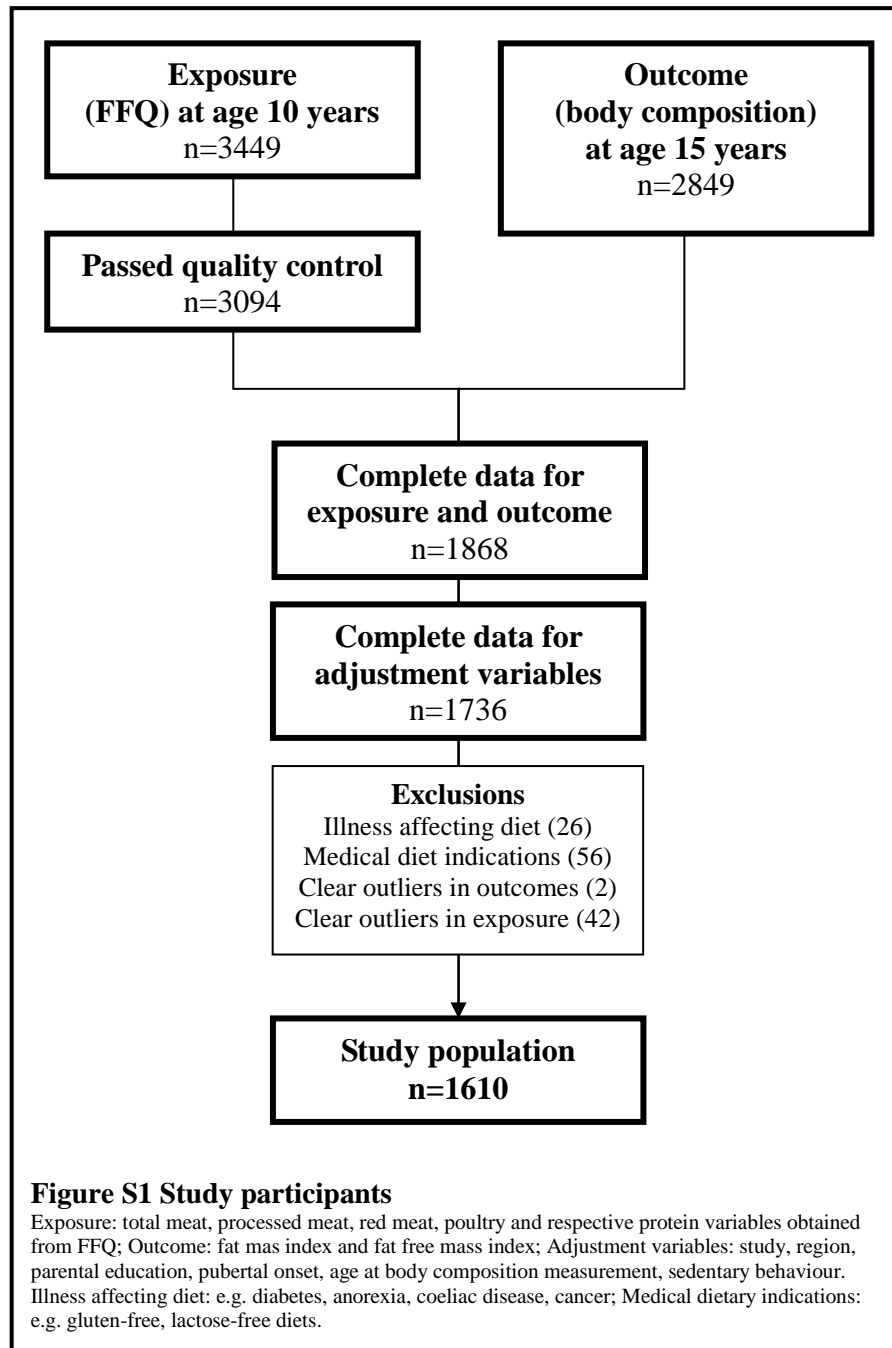

Supplement: Additional file 2: Figure S1. — Study participants. (PDF 84 kb) [file 12937_2016_222_MOESM2_ESM.pdf]
