# Supplementary material for: Prospective associations of meat consumption during childhood with measures of body composition during adolescence: results from the GINIplus and LISAplus birth cohorts
Source: Nutr J. 2016 Dec 5;15:101. doi: 10.1186/s12937-016-0222-5 (PMC5139017; doi:10.1186/s12937-016-0222-5)
Supplement: Additional file 3: Table S3a. — Prospective association of tertiles of meat and meat protein intakes with FMI and FFMI in females (n = 636) adjusted for EAA, SFA, MUFA or PUFA. Table S3b. Prospective association of tertiles of meat and meat protein intakes with FMI and FFMI in males (n = 673) adjusted for EAA, SFA, MUFA or PUFA. (PDF 272 kb) [file 12937_2016_222_MOESM3_ESM.pdf]

Table S3a Prospective association of tertiles of meat and meat protein intakes with FMI and FFMI in females (n=636) adjusted for EAA, SFA, MUFA or PUFA

	FMI						FFMI					
	T2 vsT1			T3 vsT1			T2 vsT1			T3 vsT1		
	β	SE	p-val	β	SE	p-val	β	SE	p-val	β	SE	p-val
**EAA**												
TOTAL MEAT	0.146	0.130	0.263	0.096	0.141	0.499	0.028	0.119	0.816	-0.131	0.129	0.310
PROCESSED	-0.009	0.129	0.944	0.134	0.132	0.309	0.150	0.118	0.206	0.076	0.121	0.527
RED MEAT	-0.167	0.131	0.203	-0.186	0.138	0.179	0.092	0.120	0.443	-0.154	0.126	0.224
POULTRY	-0.207	0.132	0.117	0.134	0.141	0.340	-0.197	0.121	0.105	-0.045	0.129	0.730
TOTAL MEAT PROTEIN	-0.069	0.133	0.604	0.101	0.149	0.499	-0.017	0.122	0.891	-0.174	0.136	0.202
PROCESSED (PROTEIN)	0.059	0.129	0.650	0.003	0.133	0.984	-0.018	0.118	0.877	0.089	0.122	0.465
RED MEAT (PROTEIN)	-0.139	0.131	0.290	-0.173	0.138	0.211	0.064	0.120	0.594	-0.173	0.126	0.171
POULTRY (PROTEIN)[a]	-	-	-	-	-	-	-	-	-	-	-	-
**SFA**												
TOTAL MEAT	0.172	0.130	0.186	0.251	0.132	0.058	0.050	0.119	0.674	-0.013	0.121	0.913
PROCESSED	0.023	0.130	0.862	0.240	0.133	0.071	0.169	0.119	0.154	0.141	0.121	0.243
RED MEAT	-0.115	0.131	0.379	-0.065	0.133	0.627	0.125	0.119	0.294	-0.077	0.121	0.527
POULTRY	-0.151	0.130	0.244	0.235	0.132	0.075	-0.155	0.119	0.192	0.030	0.121	0.807
TOTAL MEAT PROTEIN	-0.022	0.131	0.868	0.249	0.133	0.061	0.032	0.120	0.792	-0.033	0.121	0.784
PROCESSED (PROTEIN)	0.089	0.130	0.493	0.108	0.132	0.415	0.000	0.118	0.999	0.150	0.120	0.214
RED MEAT (PROTEIN)	-0.084	0.131	0.519	-0.054	0.133	0.684	0.099	0.119	0.404	-0.096	0.121	0.431
POULTRY (PROTEIN)[a]	-	-	-	-	-	-	-	-	-	-	-	-
**MUFA**												
TOTAL MEAT	0.198	0.132	0.134	0.295	0.148	**0.047**	0.057	0.120	0.639	-0.014	0.135	0.916
PROCESSED	0.036	0.132	0.786	0.278	0.150	0.063	0.180	0.121	0.136	0.172	0.137	0.207
RED MEAT	-0.115	0.131	0.378	-0.061	0.134	0.648	0.126	0.119	0.292	-0.072	0.122	0.553
POULTRY	-0.145	0.130	0.267	0.256	0.131	0.051	-0.149	0.119	0.211	0.046	0.120	0.701
TOTAL MEAT PROTEIN	0.001	0.132	0.996	0.284	0.140	**0.042**	0.040	0.120	0.737	-0.025	0.128	0.847
PROCESSED (PROTEIN)	0.089	0.132	0.500	0.110	0.147	0.454	0.014	0.120	0.909	0.190	0.134	0.156
RED MEAT (PROTEIN)	-0.087	0.131	0.509	-0.050	0.134	0.709	0.099	0.119	0.407	-0.091	0.122	0.457
POULTRY (PROTEIN)[a]	-	-	-	-	-	-	-	-	-	-	-	-
**PUFA**												
TOTAL MEAT	0.220	0.133	0.098	0.310	0.141	**0.029**	0.053	0.121	0.664	-0.028	0.129	0.831
PROCESSED	0.042	0.132	0.750	0.261	0.138	0.059	0.169	0.121	0.161	0.132	0.126	0.295
RED MEAT	-0.112	0.131	0.392	-0.055	0.133	0.682	0.124	0.119	0.297	-0.075	0.122	0.536
POULTRY	-0.128	0.131	0.327	0.290	0.134	**0.030**	-0.150	0.120	0.211	0.048	0.122	0.695
TOTAL MEAT PROTEIN	0.017	0.132	0.899	0.312	0.139	**0.025**	0.037	0.121	0.762	-0.036	0.128	0.780
PROCESSED (PROTEIN)	0.106	0.132	0.422	0.126	0.138	0.361	0.001	0.121	0.993	0.144	0.125	0.251
RED MEAT (PROTEIN)	-0.083	0.131	0.524	-0.043	0.133	0.745	0.097	0.119	0.415	-0.094	0.122	0.441
POULTRY (PROTEIN)[a]	-	-	-	-	-	-	-	-	-	-	-	-

EAA = essential amino acids (MAIN models for the different meat exposures additionally adjusted for energy-adjusted EAA)
SFA = saturated fatty acids (MAIN models for the different meat exposures additionally adjusted for energy-adjusted SFA)
MUFA = essential amino acids (MAIN models for the different meat exposures additionally adjusted for energy-adjusted MUFA)
PUFA = essential amino acids (MAIN models for the different meat exposures additionally adjusted for energy-adjusted PUFA)
Presented as beta coefficients (β) and standard errors (SE). Significant p-values marked in bold. [a]Estimates for poultry protein not presented as categories for protein were identical to those for poultry meat, and hence estimates are also identical.

Table S3b Prospective association of tertiles of meat and meat protein intakes with FMI and FFMI in males (n=673) adjusted for EAA, SFA, MUFA or PUFA

	FMI						FFMI					
	T2 vsT1			T3 vsT1			T2 vsT1			T3 vsT1		
	β	SE	p-val	β	SE	p-val	β	SE	p-val	β	SE	p-val
**EAA**												
TOTAL MEAT	-0.118	0.145	0.415	0.179	0.154	0.245	-0.019	0.149	0.899	0.324	0.158	**0.041**
PROCESSED	0.079	0.141	0.573	0.069	0.142	0.628	0.038	0.145	0.795	0.226	0.147	0.123
RED MEAT	-0.052	0.141	0.711	0.006	0.151	0.967	0.154	0.145	0.290	0.269	0.155	0.084
POULTRY	-0.050	0.141	0.724	0.260	0.149	0.082	-0.160	0.146	0.276	-0.082	0.155	0.596
TOTAL MEAT PROTEIN	-0.023	0.146	0.874	0.172	0.161	0.287	0.069	0.150	0.649	0.199	0.166	0.233
PROCESSED (PROTEIN)	0.033	0.140	0.815	0.106	0.143	0.457	-0.059	0.144	0.682	0.161	0.147	0.274
RED MEAT (PROTEIN)	-0.064	0.140	0.648	0.021	0.150	0.890	0.105	0.145	0.467	0.297	0.155	0.056
POULTRY (PROTEIN)[a]	-	-	-	-	-	-	-	-	-	-	-	-
**SFA**												
TOTAL MEAT	-0.084	0.138	0.543	0.249	0.141	0.077	0.034	0.143	0.812	0.427	0.145	**0.003**
PROCESSED	0.122	0.141	0.387	0.144	0.144	0.317	0.100	0.145	0.492	0.335	0.149	**0.024**
RED MEAT	-0.029	0.139	0.837	0.062	0.140	0.661	0.183	0.143	0.201	0.338	0.145	**0.020**
POULTRY	-0.037	0.139	0.791	0.270	0.139	0.053	-0.098	0.144	0.494	0.020	0.144	0.891
TOTAL MEAT PROTEIN	0.003	0.139	0.982	0.221	0.141	0.119	0.125	0.144	0.385	0.305	0.146	**0.037**
PROCESSED (PROTEIN)	0.069	0.141	0.624	0.179	0.144	0.213	-0.011	0.145	0.941	0.263	0.148	0.077
RED MEAT (PROTEIN)	-0.040	0.139	0.770	0.075	0.140	0.590	0.134	0.143	0.350	0.363	0.144	**0.012**
POULTRY (PROTEIN)[a]	-	-	-	-	-	-	-	-	-	-	-	-
**MUFA**												
TOTAL MEAT	-0.093	0.141	0.512	0.222	0.159	0.163	0.049	0.146	0.736	0.462	0.164	**0.005**
PROCESSED	0.076	0.145	0.602	0.052	0.165	0.752	0.090	0.150	0.548	0.322	0.170	0.058
RED MEAT	-0.042	0.140	0.763	0.043	0.141	0.760	0.176	0.144	0.221	0.327	0.146	**0.025**
POULTRY	-0.048	0.139	0.727	0.273	0.139	**0.050**	-0.110	0.144	0.447	0.025	0.144	0.864
TOTAL MEAT PROTEIN	-0.013	0.142	0.926	0.189	0.152	0.216	0.121	0.147	0.412	0.298	0.157	0.059
PROCESSED (PROTEIN)	0.030	0.145	0.835	0.104	0.161	0.520	-0.030	0.149	0.839	0.229	0.167	0.170
RED MEAT (PROTEIN)	-0.057	0.140	0.684	0.055	0.141	0.698	0.125	0.144	0.387	0.351	0.146	**0.016**
POULTRY (PROTEIN)[a]	-	-	-	-	-	-	-	-	-	-	-	-
**PUFA**												
TOTAL MEAT	-0.111	0.139	0.427	0.174	0.145	0.228	0.014	0.144	0.925	0.367	0.149	**0.014**
PROCESSED	0.066	0.141	0.640	0.042	0.145	0.775	0.049	0.146	0.736	0.241	0.150	0.109
RED MEAT	-0.047	0.139	0.736	0.044	0.140	0.755	0.168	0.144	0.243	0.322	0.145	**0.026**
POULTRY	-0.057	0.139	0.682	0.255	0.140	0.070	-0.121	0.144	0.401	0.003	0.146	0.983
TOTAL MEAT PROTEIN	-0.026	0.140	0.854	0.161	0.146	0.269	0.101	0.145	0.488	0.255	0.151	0.091
PROCESSED (PROTEIN)	0.015	0.141	0.915	0.087	0.144	0.546	-0.062	0.146	0.672	0.175	0.149	0.241
RED MEAT (PROTEIN)	-0.063	0.139	0.649	0.055	0.140	0.694	0.114	0.144	0.427	0.345	0.144	**0.017**
POULTRY (PROTEIN)[a]	-	-	-	-	-	-	-	-	-	-	-	-

EAA = essential amino acids (MAIN models for the different meat exposures additionally adjusted for energy-adjusted EAA)
SFA = saturated fatty acids (MAIN models for the different meat exposures additionally adjusted for energy-adjusted SFA)
MUFA = essential amino acids (MAIN models for the different meat exposures additionally adjusted for energy-adjusted MUFA)
PUFA = essential amino acids (MAIN models for the different meat exposures additionally adjusted for energy-adjusted PUFA)
Presented as beta coefficients (β) and standard errors (SE). Significant p-values marked in bold. [a]Estimates for poultry protein not presented as categories for protein were identical to those for poultry meat, and hence estimates are also identical.
