# Supplementary material for: Prospective associations of meat consumption during childhood with measures of body composition during adolescence: results from the GINIplus and LISAplus birth cohorts
Source: Nutr J. 2016 Dec 5;15:101. doi: 10.1186/s12937-016-0222-5 (PMC5139017; doi:10.1186/s12937-016-0222-5)
Supplement: Additional file 4: Table S2a. — Prospective association of tertiles of meat and meat protein intakes (T2 and T3 vs T1) with FMI and FFMI, stratified by BMI at 10y (normal weight/overweight), in females. Table S2b. Prospective association of tertiles of meat and meat protein intakes (T2 and T3 vs T1) with FMI and FFMI, stratified by BMI at 10y (normal weight/overweight), in males. (PDF 248 kb) [file 12937_2016_222_MOESM4_ESM.pdf]

**Table S2a** Prospective association of tertiles of meat and meat protein intakes (T2 and T3 vs T1) with FMI and FFMI, stratified by BMI at 10y (normal weight/overweight), in females

| | **FMI** | | | | | | | | | **FFMI** | | | | | | | | |
|---|---|---|---|---|---|---|---|---|---|---|---|---|---|---|---|---|---|---|
| | **T2 vsT1** | | | | **T3 vs T1** | | | | | **T2 vs T1** | | | | **T3 vs T1** | | | | |
| | β | SE | p-val | p-int | β | SE | p-val | p-int | p-trend | β | SE | p-val | p-int | β | SE | p-val | p-int | p-trend |
| **TOTAL MEAT** | | | | | | | | | | | | | | | | | | |
| Normal weight | 0.167 | 0.124 | 0.180 | 0.365 | 0.187 | 0.125 | 0.135 | 0.487 | 0.135 | 0.136 | 0.128 | 0.289 | 0.563 | 0.091 | 0.129 | 0.482 | 0.392 | 0.481 |
| Overweight | 0.503 | 0.469 | 0.285 | | 0.271 | 0.478 | 0.571 | | 0.547 | -0.375 | 0.310 | 0.229 | | -0.137 | 0.316 | 0.665 | | 0.634 |
| **PROCESSED** | | | | | | | | | | | | | | | | | | |
| Normal weight | 0.090 | 0.124 | 0.466 | 0.525 | 0.136 | 0.124 | 0.273 | 0.135 | 0.273 | 0.265 | 0.127 | 0.037 | **0.097** | 0.181 | 0.127 | 0.155 | 0.236 | 0.152 |
| Overweight | 0.028 | 0.470 | 0.953 | | 0.816 | 0.478 | 0.090 | | 0.092 | -0.220 | 0.316 | 0.487 | | -0.127 | 0.321 | 0.692 | | 0.686 |
| **RED MEAT** | | | | | | | | | | | | | | | | | | |
| Normal weight | -0.046 | 0.125 | 0.712 | 0.378 | -0.007 | 0.126 | 0.957 | 0.908 | 0.955 | 0.165 | 0.128 | 0.195 | 0.187 | 0.010 | 0.130 | 0.941 | 0.558 | 0.935 |
| Overweight | -0.668 | 0.482 | 0.168 | | -0.312 | 0.494 | 0.528 | | 0.523 | -0.234 | 0.319 | 0.465 | | -0.459 | 0.327 | 0.164 | | 0.162 |
| **POULTRY** | | | | | | | | | | | | | | | | | | |
| Normal weight | -0.019 | 0.125 | 0.881 | **0.016** | 0.314 | 0.125 | **0.012** | 0.481 | **0.012** | -0.100 | 0.129 | 0.439 | 0.132 | 0.102 | 0.129 | 0.429 | 0.525 | 0.426 |
| Overweight | -0.085 | 0.478 | 0.859 | | 0.026 | 0.468 | 0.955 | | 0.960 | 0.154 | 0.316 | 0.628 | | -0.124 | 0.309 | 0.689 | | 0.702 |
| | | | | | | | | | | | | | | | | | | |
| **TOTAL MEAT PROTEIN** | | | | | | | | | | | | | | | | | | |
| Normal weight | -0.034 | 0.125 | 0.788 | 0.718 | 0.154 | 0.127 | 0.224 | 0.209 | 0.224 | 0.042 | 0.129 | 0.743 | 0.172 | -0.006 | 0.131 | 0.961 | 0.529 | 0.961 |
| Overweight | 0.196 | 0.488 | 0.689 | | -0.109 | 0.477 | 0.820 | | 0.826 | -0.335 | 0.319 | 0.296 | | -0.576 | 0.312 | 0.067 | | 0.066 |
| **PROCESSED (PROTEIN)** | | | | | | | | | | | | | | | | | | |
| Normal weight | 0.111 | 0.124 | 0.372 | 0.268 | 0.071 | 0.124 | 0.570 | 0.390 | 0.566 | 0.117 | 0.127 | 0.357 | **0.016** | 0.230 | 0.128 | 0.071 | 0.111 | 0.071 |
| Overweight | -0.316 | 0.473 | 0.505 | | 0.304 | 0.485 | 0.532 | | 0.570 | -0.291 | 0.314 | 0.356 | | -0.311 | 0.322 | 0.335 | | 0.321 |
| **RED MEAT (PROTEIN)** | | | | | | | | | | | | | | | | | | |
| Normal weight | -0.010 | 0.124 | 0.937 | 0.337 | -0.036 | 0.126 | 0.778 | 0.662 | 0.778 | 0.208 | 0.127 | 0.103 | 0.198 | -0.037 | 0.129 | 0.775 | 0.601 | 0.789 |
| Overweight | -0.668 | 0.482 | 0.168 | | -0.312 | 0.494 | 0.528 | | 0.523 | -0.234 | 0.319 | 0.465 | | -0.459 | 0.327 | 0.164 | | 0.162 |
| **POULTRY (PROTEIN)[a]** | | | | | | | | | | | | | | | | | | |
| Normal weight | - | - | - | - | - | - | - | - | - | - | - | - | - | - | - | - | - | - |
| Overweight | - | - | - | | - | - | - | | | - | - | - | | - | - | - | | |

Presented as beta coefficients (β) and standard errors (SE). Normal weight: BMI z-score ≤ 1; Overweight: BMI z-score > 1. P-val: p-value for the stratified model coefficients. Significant p-values marked in bold (<0.025 after adjustment for multiple testing). P-int: P-value for the interaction term coefficients of the interaction model (p<0.1 is marked as statistically significant). p-val: p-value from linear regression; p-trend: p-value indicating linear trend. Significant p-values marked in bold.

[a]Estimates for poultry protein not presented as categories for protein were identical to those for poultry meat, and hence estimates are also identical.

**Table S2b** Prospective association of tertiles of meat and meat protein intakes (T2 and T3 vs T1) with FMI and FFMI, stratified by BMI at 10y (normal weight/overweight), in males

| | **FMI** | | | | | | | | | **FFMI** | | | | | | | | |
|---|---|---|---|---|---|---|---|---|---|---|---|---|---|---|---|---|---|---|
| | **T2 vsT1** | | | | **T3 vs T1** | | | | | **T2 vs T1** | | | | **T3 vs T1** | | | | |
| | β | SE | p-val | p-int | β | SE | p-val | p-int | p-trend | β | SE | p-val | p-int | β | SE | p-val | p-int | p-trend |
| **TOTAL MEAT** | | | | | | | | | | | | | | | | | | |
| Normal weight | -0.008 | 0.132 | 0.951 | 0.365 | 0.188 | 0.131 | 0.153 | 0.979 | 0.153 | 0.146 | 0.150 | 0.333 | **0.027** | 0.350 | 0.150 | **0.020** | 0.823 | **0.020** |
| Overweight | -0.076 | 0.426 | 0.859 | | 0.343 | 0.424 | 0.419 | | 0.416 | -0.386 | 0.374 | 0.303 | | 0.488 | 0.372 | 0.191 | | 0.191 |
| **PROCESSED** | | | | | | | | | | | | | | | | | | |
| Normal weight | 0.178 | 0.132 | 0.178 | 0.394 | 0.061 | 0.132 | 0.645 | 0.437 | 0.652 | 0.138 | 0.151 | 0.360 | 0.222 | 0.325 | 0.151 | 0.031 | 0.480 | 0.031 |
| Overweight | -0.296 | 0.426 | 0.487 | | 0.287 | 0.419 | 0.494 | | 0.488 | -0.182 | 0.379 | 0.633 | | 0.100 | 0.374 | 0.790 | | 0.785 |
| **RED MEAT** | | | | | | | | | | | | | | | | | | |
| Normal weight | -0.085 | 0.132 | 0.523 | 0.505 | -0.053 | 0.133 | 0.692 | 0.114 | 0.690 | 0.186 | 0.151 | 0.217 | 0.764 | 0.319 | 0.151 | 0.035 | 0.744 | 0.035 |
| Overweight | 0.120 | 0.431 | 0.782 | | 0.450 | 0.434 | 0.301 | | 0.298 | 0.101 | 0.382 | 0.793 | | 0.433 | 0.385 | 0.263 | | 0.259 |
| **POULTRY** | | | | | | | | | | | | | | | | | | |
| Normal weight | 0.048 | 0.132 | 0.716 | 0.980 | 0.205 | 0.132 | 0.122 | 0.593 | 0.123 | -0.127 | 0.151 | 0.403 | 0.905 | -0.084 | 0.152 | 0.581 | 0.239 | 0.578 |
| Overweight | 0.304 | 0.441 | 0.491 | | 0.133 | 0.438 | 0.762 | | 0.776 | 0.094 | 0.392 | 0.811 | | 0.033 | 0.389 | 0.933 | | 0.938 |
| | | | | | | | | | | | | | | | | | | |
| **TOTAL MEAT PROTEIN** | | | | | | | | | | | | | | | | | | |
| Normal weight | 0.002 | 0.132 | 0.988 | 0.350 | 0.189 | 0.133 | 0.156 | 0.623 | 0.156 | 0.104 | 0.151 | 0.491 | 0.580 | 0.305 | 0.152 | 0.044 | 0.584 | 0.044 |
| Overweight | -0.327 | 0.437 | 0.455 | | 0.097 | 0.437 | 0.825 | | 0.816 | -0.170 | 0.389 | 0.662 | | 0.093 | 0.389 | 0.811 | | 0.805 |
| **PROCESSED (PROTEIN)** | | | | | | | | | | | | | | | | | | |
| Normal weight | 0.131 | 0.132 | 0.324 | 0.167 | 0.037 | 0.132 | 0.782 | 0.602 | 0.788 | 0.057 | 0.151 | 0.704 | **0.092** | 0.272 | 0.151 | 0.072 | 0.500 | 0.072 |
| Overweight | -0.278 | 0.429 | 0.518 | | 0.312 | 0.423 | 0.462 | | 0.457 | -0.315 | 0.382 | 0.411 | | -0.070 | 0.377 | 0.854 | | 0.859 |
| **RED MEAT (PROTEIN)** | | | | | | | | | | | | | | | | | | |
| Normal weight | -0.083 | 0.132 | 0.528 | 0.520 | -0.031 | 0.133 | 0.813 | **0.078** | 0.811 | 0.166 | 0.151 | 0.272 | 0.690 | 0.304 | 0.151 | 0.045 | 0.689 | 0.045 |
| Overweight | 0.196 | 0.427 | 0.647 | | 0.488 | 0.431 | 0.259 | | 0.257 | 0.169 | 0.379 | 0.656 | | 0.467 | 0.382 | 0.224 | | 0.222 |
| **POULTRY (PROTEIN)[a]** | | | | | | | | | | | | | | | | | | |
| Normal weight | - | - | - | - | - | - | - | - | - | - | - | - | - | - | - | - | - | - |
| Overweight | - | - | - | | - | - | - | | | - | - | - | | - | - | - | | |

Presented as beta coefficients (β) and standard errors (SE). Normal weight: BMI z-score ≤ 1; Overweight: BMI z-score > 1. P-val: p-value for the stratified model coefficients. Significant p-values marked in bold (<0.025 after adjustment for multiple testing). P-int: P-value for the interaction term coefficients of the interaction model (p<0.1 is marked as statistically significant). p-val: p-value from linear regression; p-trend: p-value indicating linear trend. Significant p-values marked in bold.

[a]Estimates for poultry protein not presented as categories for protein were identical to those for poultry meat, and hence estimates are also identical.
